# Predicting Coordination Variability of Selected Lower Extremity Couplings during a Cutting Movement: An Investigation of Deep Neural Networks with the LSTM Structure

**DOI:** 10.3390/bioengineering9090411

**Published:** 2022-08-23

**Authors:** Enze Shao, Qichang Mei, Jingyi Ye, Ukadike C. Ugbolue, Chaoyi Chen, Yaodong Gu

**Affiliations:** 1 Faculty of Sports Science, Ningbo University, Ningbo 315211, China; 2 Auckland Bioengineering Institute, The University of Auckland, Auckland 1010, New Zealand; 3 School of Health and Life Sciences, University of the West of Scotland, South Lanarkshire, Hamilton G72 0LH, UK

**Keywords:** cutting movement, vector coding technique, inertial sensor, deep neural network

## Abstract

There are still few portable methods for monitoring lower limb joint coordination during the cutting movements (CM). This study aims to obtain the relevant motion biomechanical parameters of the lower limb joints at 90°, 135°, and 180° CM by collecting IMU data of the human lower limbs, and utilizing the Long Short-Term Memory (LSTM) deep neural-network framework to predict the coordination variability of selected lower extremity couplings at the three CM directions. There was a significant (*p* < 0.001) difference between the three couplings during the swing, especially at 90° vs the other directions. At 135° and 180°, t13-he coordination variability of couplings was significantly greater than at 90° (*p* < 0.001). It is important to note that the coordination variability of Hip rotation/Knee flexion-extension was significantly higher at 90° than at 180° (*p* < 0.001). By the LSTM, the CM coordination variability for 90° (CMC = 0.99063, RMSE = 0.02358), 135° (CMC = 0.99018, RMSE = 0.02465) and 180° (CMC = 0.99485, RMSE = 0.01771) were accurately predicted. The predictive model could be used as a reliable tool for predicting the coordination variability of different CM directions in patients or athletes and real-world open scenarios using inertial sensors.

## 1. Introduction

After the central motor nervous system receives a command to change direction, the body completes the cutting movements (CM). This is a common movement process, particularly in team sports such as basketball and soccer [1,2,3]. Additionally, it is a common technique and tactic for athletes to use CM to deceive their opponents during the competition [4]. Therefore, individuals or teams execute effective CM amongst themselves, which is crucial for both training and competitions [4,5,6]. Moreover, the completion of a successful CM is a means of promoting positive results in competitions and a criterion for selecting athletic talent [7].

In order to perform high-quality CM, the body conducts deceleration and acceleration movements over brief intervals, generating high physiological and mechanical loads [8]. In particular, the knee valgus moment was higher during 90°, 135° and 180° CM in men [9]. This may result in non-contact ligament damage to the knee and ankle joints [2,10,11,12], such as anterior cruciate ligament injury of the knee [13], strain of the medial collateral ligament of the meniscus [14] and ankle sprains [15]. In addition to affecting the knee and ankle joints, CM places high demands on hip-joint function [16], and requires athletes to be able to strongly extend their hips and withstand higher ground reaction forces. Research shows [17,18], the efficient use of the hip joint during CM is crucial for relieving knee pain and enhancing the lower extremity function. Notably, to perform body deceleration braking and trunk redirection acceleration, an effective transition mechanism is required to prevent sports injuries and achieve high-quality CM [19]. To accomplish this, such a transition mechanism requires coordination between the joints of the lower limbs [20,21]. Additionally, monitoring and adjusting the range of motion between joints effectively prevent sports injuries [22,23,24,25]. Collaboration between the hip, knee and ankle joints will promote dynamic stabilization and force generation in the legs, thereby optimizing the CM transition mechanism [26]. Unfortunately, previous researchers have overlooked the variability in the strategies and changes in movement patterns associated with CM [12].

Inter-joint coordination is a comparison of the relationship between two joint movements, requiring the collection and scientific quantification of data from the corresponding joints during the motion. The three primary methods that evaluate the coordination and coordination variability of coupling behaviors are [27]: (1) discrete relative phase; (2) continuous relative phase, and; (3) vector coding. The discrete relative phase is often used to evaluate the timing of key events in each of the angular profiles [28]. The relative phase diagram approach combines information on joint angular position and velocity, which is used to gain insight into the behavior of non-linear, limit cycle oscillatory systems in engineering [29]. As a well-established technique for quantifying joint-coordination patterns [30], the vector coding technique utilizes angle-angle maps to estimate coordination measures and thus evaluate coordination variability.

Researchers typically rely on 3D motion-analysis systems and force plates to collect kinematic and kinetic data on participants for measuring individual lower extremity joint coordination [8]. However, factors such as high laboratory costs and limited spatial implementation prevent researchers from assessing the motor behaviors of populations such as patients and athletes in clinical and exercise settings [31,32]. The wearable device can more directly depict the biomechanical traits of the athlete in an actual situation than a lab test, which is helpful for biomechanical analysis of complex movements [33,34]. The inertial Measurement Unit (IMU), a sensor that combines gyroscopes, accelerometers, and magnetometers, is the primary tool for quantifying motion behavior [35]. Its benefits include portability and wearability (via wireless transmission of real-time data), ease of operation, simplicity of analysis, a wide range of application scenarios (underwater use with sealed settings), energy efficiency (for long-term measurement work, such as marathon monitoring), and adequate measurement range and sampling frequency. Simultaneously, it has some technical issues, such as the lack of fit between the body and the device during exercise, resulting in soft tissue artefacts due to shock during the test, which may compromise the data’s accuracy [36]. Second, ferromagnetic disturbances can also result in outdoor measurement errors [37]. In order to overcome the technical issues of IMU and improve the data’s reliability, numerous studies have applied machine learning techniques to reduce the IMU data’s acquisition error [38,39,40]. This type of method utilizes IMU-collected data (as input features) to train and validate an algorithm that generates accurate predictions for new data input [41].

Zago et al. [42] used machine learning and wearable sensors to predict the energy and motion of CM, while a neural network structure method gave accurate results. Previous research [43,44] investigated the coordination variability of the CM using a vector-coding technique. Using laboratory equipment, we were unable to make real-time measurements and calculations while athletes performed CM in an open environment. Consequently, our research aims to use wearable sensors and combine them with a deep-learning model, which will attempt to predict the coordination variability under three CM directions. By examining the data on inter-articular coordination variability, the control of the locomotor system during CM can be better understood. This information will support the creation of assessment protocols for the motor rehabilitation of patients with ACL tears, ankle sprains, etc., [34].

## 2. Materials and Methods

### 2.1. Participants

We recruited 25 healthy male college students (all participants were male, age: 23 ± 2 years, height: 1.76 ± 0.05 m, weight: 70.62 ± 4.74 kg) from Ningbo University [9] who exercised at least three times per week for 45 min on average per session. Before data collection, participants were familiar with the CM experimental procedure. According to the physician’s assessment, all participants had no exercise restrictions and a normal body-mass index. There was no lower extremity or back musculoskeletal discomfort or injury lasting more than one week, six months before participation in the trial, no history of lower extremity or back surgery, and no current usage of foot orthotics. Ningbo University’s ethics review committee approved this work (RAGH202203012707). Before participating, all participants completed a written informed consent form.

### 2.2. Instruments

**Figure 1:**
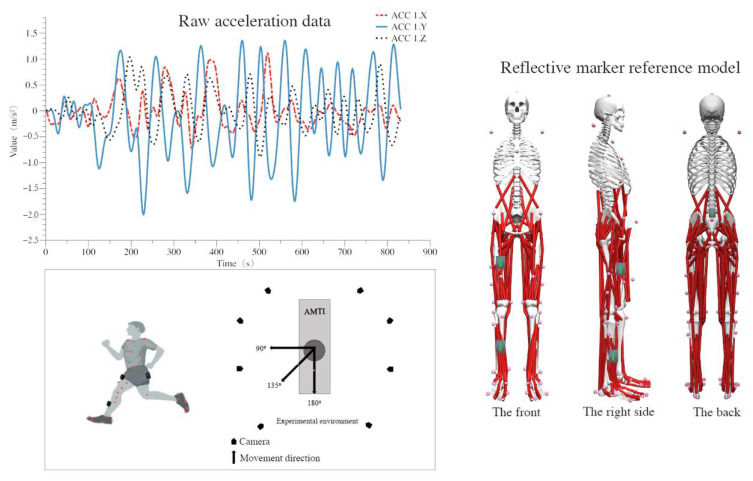
The sensor’s raw data and the diagram of reflective markers.

The Vicon 3D analysis system (Vicon Metrics Ltd., Oxford, UK) with eight infrared cameras was utilized to acquire kinematic data of the lower limbs at 200 Hz. Vertical ground reaction forces were measured using AMTI force plates (AMTI, Watertown, MA, USA) at 1000 Hz. The CM’s speed was monitored during the test using a speed measurement device (Smart speed, Fusion Sport Inc., Burlingame, CA, United States). Three inertial sensors (Delsys Inc., Natick, MA, USA) were attached to the anterior calf, anterior thigh, and L6 Vertebra, locations often utilized in portable testing [35]. Each participant was fitted with 39 (12.5 mm diameter) reflective markers following the Opensim (Stanford University, Stanford, CA, USA) Gait 2392 model [45]. Optical reflection markers were connected to each participant’s anatomical landmarks in accordance with the Gait 2392 model, and the placement of the markers was always performed by the same operator. Figure 1 depicts the location of each marker during 3D motion capture.

### 2.3. Procedures

For the testing, each participant wore matching, form-fitting shorts and shoes. Under the guidance of a professional physical trainer, a 10-min warm-up was conducted. Prior to beginning the formal experiment, the participant was permitted three practice runs to familiarize himself with the test procedures. The reflex markers and portable sensors were set up precisely in the appropriate positions for each participant. The sensors are attached directly to the skin with double-sided surgical tape and held in place with nylon straps, which reduce data errors by minimizing soft tissue movement [46].

Each participant was instructed to stand parallel to the Y-axis of the force platform with their hands naturally spread, palms facing forward, and looking forward until the complete static pose was captured. Participants were subjected to a 6 m CM test to collect kinematic and kinetic data in the laboratory. Before each test, the subject was instructed to remain stationary at the starting position for three seconds (to facilitate control of the accelerometer and calibration of the gyroscope to capture the start/stop phase of the direction of motion). Participants accelerated by running on a 6-m track, placing their right foot (with a heel-first hitting the ground strategy) entirely on the force platform for braking, and then completing 90°, 135°, and 180° CM turns at the angles indicated by the landmarks (Figure 1). In order to avoid the effect of proficiency on coordination [47], participants were instructed not to perform two consecutive CMs in the same direction. In addition, the participant must maintain a speed between 3.5 m/s and 6.5 m/s for each CM [48]. All participants were required to implement the CM test three times in different directions (9 practical experiments per participant). In order to avoid the effects of fatigue on the variability of lower-limb joint coordination, participants were given a 1-min break following each CM test [49].

### 2.4. Data Processing

#### 2.4.1. Initial Data Processing

The Vicon Nexus 1.8.6 software was used to capture kinematic biomechanical parameters and exporting error-free data to c3d format files. Kinematic and ground reaction force data underwent coordinate system conversion, low-pass filtering, data extraction, and format conversion using MATLAB R2018b (The MathWorks, Natick, MA, USA). It was filtered using a 6-Hz and 30-Hz fourth-order, zero-phase lag Butterworth low-pass filter for marker trajectories and ground reaction forces. The Gait 2392 musculoskeletal model in Opensim (Stanford University, Stanford, CA, USA) was used to calculate kinematic and inverse kinetic parameters. The angle data were linearly interpolated to 101 data points, with each point representing 1% of the stance phase (0–100%).

As an input feature, the stance phase of the right foot during CM was extracted from the inertial sensor (Delsys Inc., Natick, MA, USA), as shown in Figure 1. Due to interference from the field environment, the magnetometer data could not be utilized during data selection for this experiment. Therefore, a total of 18 input features were collected. Smoothing of data was achieved with a 15 Hz low-pass filter. The dataset was normalized so that all the values were within the range 0–1, which was the way the neural network architecture was adapted [50,51].

#### 2.4.2. A Quantitative Approach to Coordination

There are various techniques for quantifying coordination variability [52], but the techniques chosen should be based on the research questions. When participants perform CM, the joint movements of their lower extremities exhibit a non-sinusoidal pattern (except for the sagittal plane movements of the hip joint) [53]. However, Vector coding permits tests with non-sinusoidal motion patterns and is therefore deemed more suitable for clinical testing [49,54]. This technique was used in this study to quantify coordination variability of selected lower extremity couplings: Thigh abduction-adduction/ Leg flexion-extension (Thigh A/A-Leg F/E); Hip rotation /Knee flexion-extension (Hip R-Knee F/E); Knee flexion-extension/Ankle rotation (Knee F/E-Ankle R). These couplings are the focal points of patellofemoral joint-pain syndrome examination [48].

Using the vector-coding technique developed by Tepavac and Field-Fote and referencing the interpretation of vector coding by Samaan MA et al. (2015) and Tepavac, D et al. (2001) [49,55], we determined the joint coordination variability in this study. The calculations were performed with a custom MATLAB program (see the Appendix A for details of the calculation procedure).

### 2.5. Long Short Term Memory (LSTM) Network Algorithm Model

In this study, the 18 extracted features from the three IMU data exhibit properties such as high nonlinearity and continuity, which are consistent with the analysis characteristics of the recurrent neural network (RNN) models [56]. The RNN is an algorithmic model devoted to predicting highly nonlinear data on time series, which introduces the concept of neuronal networks to imitate human memory [57]. As an extension of the RNN algorithm, Long Short-Term Memory (LSTM) has been used. It solves the gradient vanishing problem in RNN algorithms, which can be trained to ensure that the gradient of the objective function to the state signal does not entirely vanish [58].

The fundamental LSTM architecture includes two internal cell states, namely the hidden layer state (h_t−1_) and the cell state (C_t−1_), as shown in the equation below. (Equations (1)–(6))

(1) $$f_{t}=\sigma_{\mathrm{sigmoid}}\;\left(W_{f\;}\left[h_{t-1},x_{t}\right]+b_{f}\right)$$

(2) $$i_{t}=\sigma_{\mathrm{sigmoid}}\;\left(W_{i\;}\left[h_{t-1},x_{t}\right]+b_{i}\right)$$

(3) $$\tilde{C_{t}}=\tan h\left(W_{C}\;\left[h_{t-1},x_{t}\right]+b_{C}\right)$$

(4) $$C_{t}=f_{t}\times C_{t-1}+i_{t}\times \tilde{C_{t}}$$

(5) $$o_{t}=\sigma_{\mathrm{sigmoid}\;}\left(W_{o\;}\left[h_{t-1},x_{t}\right]+b_{o}\right)$$

(6) $$h_{t}=o_{t}\times \tan h\left(C_{t}\right)$$

**Figure 2:**
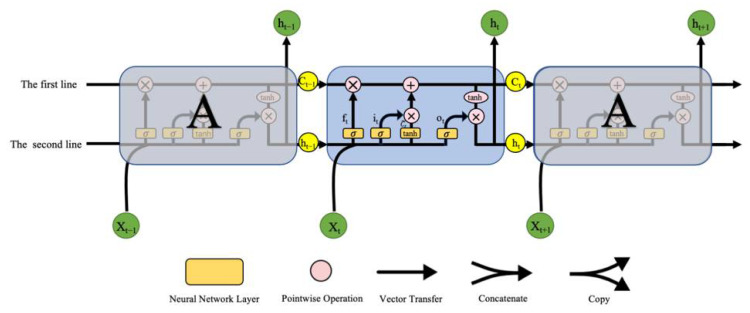
Schematic representation of the computational process for the fundamental LSTM structure.

Time is represented by the symbol t in the equation. At time t, the LSTM architecture generates inputs and outputs. There are three inputs: the cell state C_t−1_, the hidden state h_t−1_, and the vector value x_t_ of the input at time t. The basic framework has two outputs: the cell state C_t_ and the hidden state h_t_. The activation function σ_sigmoid_ is employed. f_t_ is used as the forgetting gate’s value. The update gate’s output value (C_t_) consists of two components: i_t_ and $\tilde{C_{t}}$. tanh is an activation function that normalizes input values to −1 to 1. Together, Equations (5) and (6) constitute the calculation for the output gates. W_f_, W_i_, W_C_, W_o_ and b_f_, b_i_, b_C_, b_o_, respectively, represent the weights and bias variables of the three gates and a storage cell. The cell state C_t−1_ will always be in the transmitted state, as shown in Figure 2, which is a schematic representation of the LSTM structure’s computation procedure. At this time, the hidden state h_t_ and the input value x_t_ are modified for C_t_ and transmitted to the next instant. The structure of the gate in the LSTM changes the information in the hidden state h_t−1_ and helps figure out the output. In general, the cell-state information will be transmitted over the first line, the hidden-state information will be transmitted over the second line, and the two lines will interact via the gate to complete the calculation.

In our study, we employ a three-layer LSTM network in our method. The input to the network is a data series, which is a time series formed after the extraction of 18 features using the sliding window method [59]. For each CM direction, 225 samples of CM were collected. Thus, the three-sensor-collected data set for each CM direction includes 675 sequence characteristics in total. The dataset was separated into a training set (70%), a validation set (20%), and a test set (10%). In addition, distinct prediction models were trained for each direction of CM (90°, 135°, 180°). The coefficient of multiple correlation (CMC) values and root mean square error (RMSE) was used to evaluate the prediction accuracy of the model [50,60], with CMC values interpreted as perfect similarity (0.95–1), very similar (0.85–0.94), moderate similarity (0.5–0.74), and poor similarity (0–0.59) [61]. RMSE values were utilized to evaluate segmental coordination prediction data, and actual data error means.

### 2.6. Statistical Analysis

Using the Shapiro-Wilk test (SPSSs Inc., Chicago, IL, USA), a normal distribution of the coordination variability and the vertical ground reaction forces in different CM directions was determined. If the normal distribution was satisfied, a one-way repeated measures ANOVA with one-dimensional statistical parametric mapping (SPM1d) was performed. If the distribution is not normal, a one-way repeated-measures ANOVA with one-dimensional statistical nonparametric mapping (SnPM1d) is performed [62]. In the case of significant main effects (directions), Bonferroni adjustment was used to post hoc paired comparisons of significant main effects (directions). SPM1d and SnPM1d were statistically analyzed using a MATLAB open-source script (The MathWorks, Natick, MA, USA). The significance level for each test was set to 0.05.

## 3. Results

### 3.1. Shapiro-Wilk

Using Shapiro-Wilk tests, the normality of the vector coding and vertical ground reaction forces under the three CM directions was determined. Initial hypotheses suggested that the sample did not significantly deviate from the normal distribution, i.e., it conformed to the normal distribution. At the significance level of 0.05, (*p* < 0.05), the original hypothesis was rejected, and none of the samples conformed to the normal distribution (Table 1). Consequently, we employed SnPM1d for statistical analysis.

### 3.2. SnPM1d

**Figure 3:**
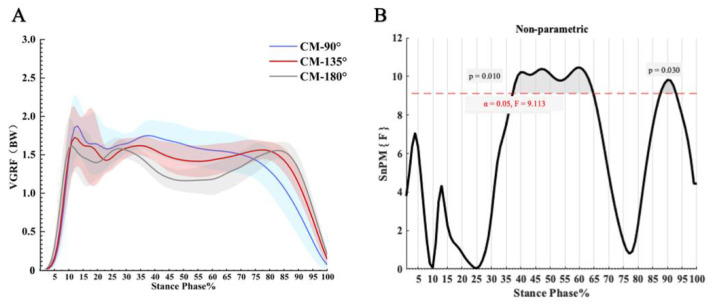
(**A**) Three CM directions of the vertical ground reaction force; (**B**) The SnPM statistics comparison.

As shown in Figure 3, the principal effect of CM direction on the vertical ground reaction force was significant and varied between 37–65% stride (*p* = 0.010) and 87–93.3% stride (*p* = 0.030), respectively. By the variation of the peak vertical ground reaction force during the CM, the CM can be roughly divided into the periods of initial contact (0–10% stride), foot flat (11–25% stride), swing (26–75% stride), and the end of the swing (76–100% stride).

**Figure 4:**
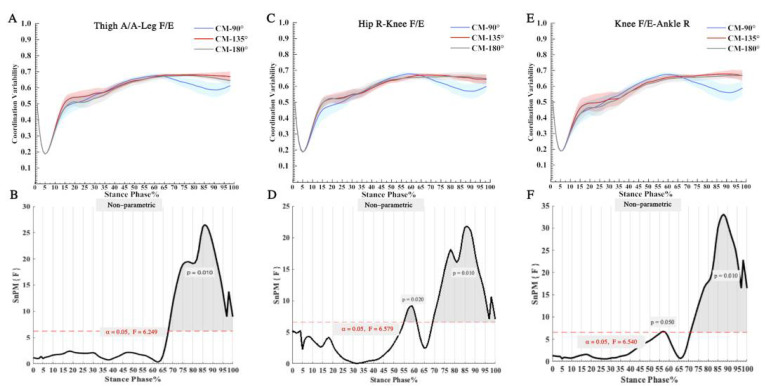
(**A**) The coordination variability of Thigh A/A-Leg F/E; (**B**) The SnPM statistics comparison of the coordination variability of Thigh A/A-Leg F/E; (**C**) The coordination variability of Hip R-Knee F/E; (**D**) The SnPM statistics comparison of the coordination variability of Hip R-Knee F/E; (**E**) The coordination variability of Knee F/E-Ankle R; (**F**) The SnPM statistics comparison of the coordination variability of Knee F/E-Ankle R.

Significant main effects were observed for Thigh A/A-Leg F/E, Hip R-Knee F/E, and Knee F/E-Ankle R on the CM direction condition (Figure 4). A significant conditional main effect (*p* = 0.010) of Thigh A/A-Leg F/E during foot swing (68–100% stride). A post hoc paired test analysis revealed (Table 2) that the coordination variability was significantly greater at 135° than it was at 90° during (68–100% stride) foot swing (*p* < 0.001). The variability of foot coordination during (72–100% stride) swing was significantly greater at 180° than at 90° (*p* < 0.001). There was no statistically significant difference between the coordination variability at 135° and 180° (*p* > 0.05).

There was a significant conditional main effect of Hip R-Knee F/E during the later stance (55–62% stride, *p* = 0.020) and foot swing of the foot (69–100% stride, *p* = 0.010), as shown in Figure 4. An analysis of post-paired tests revealed (Table 2) that the coordination variability was significantly greater at 135° than 90° during (69–91% stride) foot swing (*p* < 0.001). The coordination variability was significantly greater at 180° than at 90° during the foot swing (56–63% and 75–100% stride, *p* < 0.001). There was no statistically significant difference between the coordination variability at 135° and 180° (*p* > 0.05).

There was a significant conditional main effect (*p* = 0.010) for Knee F/E-Ankle R during foot swing (71–100% stride), as shown in Figure 4. A post hoc paired test analysis revealed (Table 2) that coordination variability was significantly higher at 135° than at 90° during (72–100% stride) foot swing (*p* < 0.001). During the (77–100% stride) foot swing, and the coordination variability was significantly greater at 180° than at 90° (*p* < 0.001). There was no statistically significant difference between coordination variability at 135° and 180° (*p* > 0.05).

### 3.3. Performance of LSTM Model

**Figure 5:**
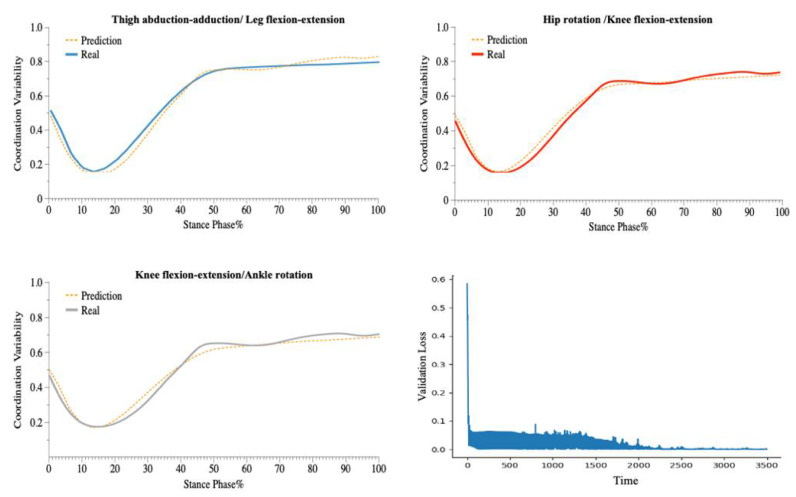
Predicted and Validation Loss Curve for the Coordination of Three Couplings during 90° CM.

**Figure 6:**
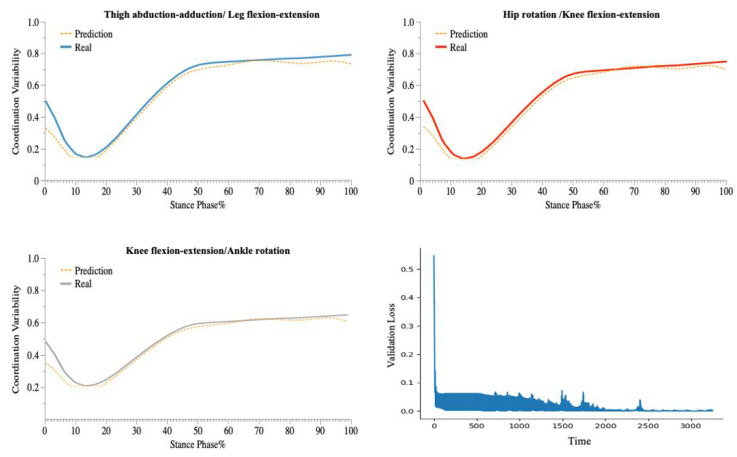
Predicted and Validation Loss Curve for the Coordination of Three Couplings during 135° CM.

**Figure 7:**
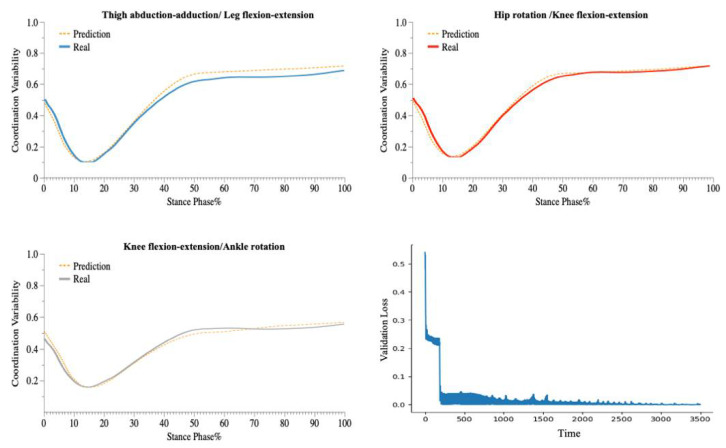
Predicted and Validation Loss Curve for the Coordination of Three Couplings during 180° CM.

The LSTM model performed well on the test set, with accurate predictions of the coordination variability of the coupling with three CM feature inputs (e.g., Figure 5, Figure 6 and Figure 7).

In the 90° CM prediction model, each layer of the LSTM contained 100 neural units. All training samples were propagated forward and backwards in the neural network 600 times (Epoch = 600), with a batch size of 1024 samples and a learning rate greater than 0.001. The model accurately predicted the direction of 90° (CMC equal to 0.99063 and RMSE equal to 0.02358).

In the 135° CM prediction model, each layer of the LSTM contained 120 neural units. All training samples were propagated forward and backwards in the neural network 700 times (Epoch = 700), with a batch size of 1024 samples and a learning rate greater than 0.001. The model accurately predicted the direction of 135° (CMC equal to 0.99018 and RMSE equal to 0.02465).

In the 180° CM prediction model, each layer of the LSTM contained 120 neural units. All training samples were propagated forward and backwards in the neural network 750 times (Epoch = 750), with a batch size of 1024 samples and a learning rate greater than 0.001. The model accurately predicted the direction of 180 ° (CMC equal to 0.99485 and RMSE equal to 0.01771).

## 4. Discussion

In this study, an improved vector-encoding technique was used to examine the variability of lower-limb coordination in various CM directions. In addition, a deep-learning prediction model, based on the LSTM structure and employing 18 sequence features captured by the IMUs, was developed. For predicting the coordination variability of the three CM directions, we constructed a deep-learning architecture with three layers of LSTM. Following the recommendation of previous research [63,64], we utilized the accelerometers’ raw acceleration data as feature data, which considerably aided our research (Figure 5, Figure 6 and Figure 7). The model predicts the coordination variability of three couplings in three CM directions with accuracy. The significance of this work is that researchers can monitor the coordination variability of patients or athletes in different CM directions using portable sensors in real-world open environments.

Researchers have used portable sensors and deep neural networks to investigate the biomechanics of lower-limb movement, which has become a popular tool for motion monitoring and functional assessment [65,66,67]. In addition, this will facilitate the efficient and accurate collection of valid feature data by researchers. In previous research, the disadvantage of simple machine-learning models was that they could only fit predictions to linear data with apparent features and was susceptible to generalized predictive error [68]. However, deep neural networks effectively learn nonlinear relationships in high-dimensional data. They can train and predict dense time-series data collected by IMU with efficiency and precision [69]. This study incorporated sensor data from three IMU as model features. In terms of data performance, it was demonstrated that the LSTM deep neural network model could achieve outstanding performance with a relatively small data set [70].

In terms of observing the coordination variability between the lower limb couplings during CM, our results are consistent with other studies (within expectation CM) [44] and demonstrates a high degree of coordination variability in each of the three CM directions. Using the SnPM1d, we compared the disparity between the coordination variability of the three CM directions. In addition, by observing the peak variation of the vertical ground reaction force during the CM, we approximated the period of initial contact (0–10% stride), the period of the flat-footed foot flat (11–25% stride), the period of the swing (26–75% stride), and the end of the swing (75–100% stride). The coordinated variability of all three couplings varied significantly between 0% and 25% stride (Figure 4), indicating that the body must deal with stress loads during braking, and conduct a high level of coordinated action between the joints. The “U” pattern of “high-low-high” with its complex coordination variability may result in the overuse of the lower extremity couplings, causing chronic movement injuries in the lower extremity joints [48]. Notably, we did not find coordinated deformable differences in the three different directions between 0% and 25% stride (e.g., Figure 4), so it can be assumed that whatever direction of CM may all have a high risk of injury during this period [71,72]. In addition, during CM in heel-strike mode, the knee joint plays a significant role, and the absorbed energy is redistributed and absorbed by the knee extensors [73]. In light of this, the functionality of the knee joint is crucial at such high mechanical intensities.

In this study, the variability of the three couplings during foot swing varied significantly, particularly in the 90° direction compared to the other directions. Interestingly, there was no significant difference between 135° and 180° in lower limb coupling coordination variability. At the beginning of the swing phase, approximately 70% of the stride, a decreasing trend in coordination variability was observed for the 90° CM in the Thigh A/A-Leg F/E, Hip R-Knee F/E, and Knee F/E-Ankle R tests. Consequently, the coordination variability of all three couplings was significantly greater at 135° and 180° than it was at 90° (*p* < 0.001). This may be because the 135° and 180° CM require a more urgent completion of the motor task per unit time, resulting in a coordination variability that persists at this stage. In addition, since the 90° CM strategy is frequently employed in practical training, a certain level of mechanical proficiency may facilitate the completion of the task via an economical mode with low degrees of freedom [12]. Hip R-Knee F/E was discernible in the post hoc comparison of CM at 90° and 180°, with significantly greater coordination variability at 90° than at 180° (56–63% stride, *p* < 0.001). This contributes to our understanding that the high coordination variability of the coupling during 180° CM may result in postural instability and internal and external body harm [74,75]. During the initial swing phase of the 180° CM, the couplings may demonstrate instability. Pathological manifestations include delayed peak and the inability to maintain peak hip flexion during the swing phase to the stance phase [76]. For the 180° CM, we must investigate further the biomechanical characteristics of the initial phase of the lower-limb swing, comparing the expected and unintended cases in particular.

Previous research on the coordinated variability of different motor strategies has yielded inconsistent results [77,78]. A comparison test between professionals and amateurs determined that the “finish line” of professionals was significantly less deformed than amateurs [79]. In contrast, a study of knee injuries concluded that healthy individuals exhibited more significant coordination variability in their lower extremities, whereas individuals with knee pain exhibited lower coordination variability [22]. This appears to support our research approach for this project. The variability of different motion patterns must be incorporated into any functional motion analysis and linked to overuse injuries from a dynamic systems perspective [22]. Consequently, we focused predominantly on the various CM directions for this work’s predictive model.

These promising results must be interpreted in light of the limitations associated with these studies. First, despite using three sensors to improve the correlation between feature data and the predicted target, the validation effect may be compromised by subject-specific bias in sensor placement. In addition, the laboratory acquisition of predicted data involves multiple calculations, which imposes stringent requirements on both the laboratory acquisition and calculation processes. Future research must reduce the model’s sensitivity to sensor position bias for laboratory and pathological diagnosis. Notably, we did not sample patients or sports injury populations in this study, and only healthy males participated in this trial. Thus, acquiring features for patient or sports injury populations and women is an effective method for enhancing the model’s applicability to the population. In addition, a potential improvement direction for this work is the use of a combined deep-learning model [80]: classification of different CM directions followed by accurate predictions applicable to a wide variety of populations.

## 5. Conclusions

Using an enhanced vector-coding technique, we investigated the coordination variability of lower extremity coupling in different CM directions in this study. The results indicate that the variability of the three couplings during foot swing differs significantly between the 90° direction and the other two directions. The coordination variability was significantly greater in the 135° and 180° directions than in the 90° CM. There was no significant difference between 135° and 180° in lower-limb coupling coordination variability. In addition, we developed a deep learning prediction model using LSTM and three inertial sensors to predict the coordination variability of three lower-limb couplings. The prediction models of motion under three CM directions demonstrated excellent prediction accuracy, making it possible to replace the conventional marker-obtained data derived from 3D motion capture systems.

## Figures and Tables

**Table 1:** The Shapiro-Wilk test for normality of vector coding values and vertical ground reaction in three CM directions.

	CM Direction (°)	Significance (*p*)
	90°	0.012748
Thigh A/A-Leg F/E	135°	0.000870
	180°	0.000436
	90°	0.000119
Hip R-Knee F/E	135°	0.000002
	180°	0.000003
	90°	0.007998
Knee F/E-Ankle R	135°	0.000006
	180°	0.000227
	90°	0.000960
Vertical ground reaction force	135°	0.000756
	180°	0.000327

**Table 2:** Vector-coding coefficients, mean (standard deviation), and Post hoc test.

The Couplings	Direction (°)	Mean (SD)	Max (SD)	Post Hoc Test	
				135°	180°
Thigh A/A-Leg F/E	90°	0.564 (0.019)	0.690 (0.014)	*p* < 0.001 (68–100% stride)	*p* < 0.001 (72–100% stride)
	135°	0.588 (0.012)	0.699 (0.007)	—	*p* > 0.05
	180°	0.575 (0.012)	0.697 (0.007)	—	—
Hip R-Knee F/E	90°	0.554 (0.020)	0.688 (0.008)	*p* < 0.001 (69–91% stride)	*p* < 0.001 (56–63% stride; 75–100% stride)
	135°	0.575 (0.012)	0.697 (0.010)	—	*p* > 0.05
	180°	0.575 (0.014)	0.697 (0.020)	—	—
Knee F/E-Ankle R	90°	0.543 (0.020)	0.685 (0.008)	*p* < 0.001 (72–100% stride)	*p* < 0.001 (77–100% stride)
	135°	0.566 (0.016)	0.698 (0.006)	—	*p* > 0.05
	180°	0.555 (0.016)	0.695 (0.017)	—	—

## Data Availability

The data that support the findings of this study are available on reasonable request from the corresponding author. The data are not publicly available due to privacy or ethical restrictions.

## Appendix A

The variables x, y, and N in the following equation represent the joint-angle matrices generated during the motion of the proximal (*x*) and distal (*y*) joints, and the vector of the total number of data points (*N*).

First, it is necessary to calculate the anterior-posterior difference between the proximal and distal joints in continuous motion (Equation (A1)).

(A1) $$X_{i}=x_{i}-x_{i-1},Y_{i}=y_{i}-y_{i-1},\;\mathrm{where}\;i=1,2,\ldots N$$

Next, the vector (L) length between the corresponding points during the motion of the distal and proximal joint segments is calculated (Equation (A2)).

(A2) $$L_{i}=\sqrt{X_{i}^{2}+Y_{i}^{2}},\;\mathrm{where}\;i=1,2,\ldots N$$

At each corresponding time point, the joint kinematic data (cos*θ* and sin*θ*) angle is determined by the length of the vector between the corresponding points (Equation (A3)).

(A3) $$\cos\theta_{i}=\frac{X_{i}}{L_{i}},\;\sin\theta_{i}=\frac{Y_{i}}{L_{i}},\;\mathrm{where}\;i=1,2,\ldots N$$

The arithmetic mean is calculated for cos*θ_i_* and sin*θ_i_* in Equation (A3) (Equation (A4)).

(A4) $$\cos\theta \text{\_}mean_{i}=\frac{\sum_{i=1}^{N}\cos\theta_{i}}{N},\;\sin\theta \text{\_}mean_{i}=\frac{\sum_{i=1}^{N}\sin\theta_{i}}{N},\;\mathrm{where}\;i=1,2,\ldots N$$

Determine the average vector angle (α) by using the average cosine and sine angles (Equation (A5)).

(A5) $$\alpha_{i}=\sqrt{\cos\theta \text{\_}mean_{i}^{2}+\sin\theta \text{\_}mean_{i}^{2}},\;\mathrm{where}\;i=1,2,\ldots N$$

The arithmetic means of the average joint angle $\bar{a_{ɩ}}$ is then determined (Equation (A6)). $\bar{a_{ɩ}}$ represents the overall variation in the relationship between the two joint angles throughout all test cycles.

(A6) $$\bar{a_{ɩ}}=\frac{\sum_{i=1}^{N}\alpha_{i}}{N},\;\mathrm{where}\;i=1,2,\ldots N$$

In order to address the magnitude differences. We introduce a second parameter, m, which describes the frame-to-frame vector size variability to resolve the size disparity. We normalize the length of the frame-to-frame vector across multiple cycles by dividing all magnitudes within a given frame-to-frame interval by its greatest value (Equation (A7)).

(A7) $$l_{M}=\frac{l_{i^{\prime }}}{\max\left(l_{i^{\prime }}\right)},\;\mathrm{where}\;i^{\prime }=1,2,\ldots M$$

*M* is the cycle number. This is performed to maintain the variance below the value “1.” We will calculate the mean and standard deviation of each frame interval’s magnitude in the subsequent step. Equation (A8) computes the maximum standard deviation (*σ_max_*) for the entire data matrix, where M represents the total number of repetitions of the analyzed task.

(A8) $$\sigma_{max}=\frac{1}{2}\sqrt{\frac{2mod\left(\frac{M+1}{2}\right)-1}{2mod\left(\frac{M+1}{2}\right)}}$$

Equation (A9) calculates the magnitude deviation (m), which varies between 0 and 1.

(A9) $$m_{i}=1-\left(\frac{\sigma l_{M}}{\sigma_{max}}\right)=1-\frac{1}{\sigma_{max}}\sqrt{\frac{1}{M-1}\sum_{i=0}^{M-1}(l_{M}-\bar{a_{i}}{)}^{2}},\;\mathrm{where}\;i=1,2,\ldots N$$

The joint coordination variability *V* was calculated using Equation (A10), a method proposed by Tepavac et al.

(A10) $$V_{Tep}=\alpha_{i}\times m_{i},\;\mathrm{where}\;i=1,2,\ldots N$$

Equation (A11) determines the magnitude of joint coordination variability, with “0” representing no deformability and “1” representing high deformability.

(A11) $$V=1-V_{Tep}$$
